# Modelling and measuring single cell RNA expression levels find considerable transcriptional differences among phenotypically identical cells

**DOI:** 10.1186/1471-2164-9-268

**Published:** 2008-06-03

**Authors:** Tatiana Subkhankulova, Michael J Gilchrist, Frederick J Livesey

**Affiliations:** 1 Gurdon Institute and Department of Biochemistry, University of Cambridge, Tennis Court Road, Cambridge, CB2 1 QN, UK

## Abstract

**Background:**

Phenotypically identical cells demonstrate predictable, robust behaviours. However, there is uncertainty as to whether phenotypically identical cells are equally similar at the underlying transcriptional level or if cellular systems are inherently noisy. To answer this question, it is essential to distinguish between technical noise and true variation in transcript levels. A critical issue is the contribution of sampling effects, introduced by the requirement to globally amplify the single cell mRNA population, to observed measurements of relative transcript abundance.

**Results:**

We used single cell microarray data to develop simple mathematical models, ran Monte Carlo simulations of the impact of technical and sampling effects on single cell expression data, and compared these with experimental microarray data generated from single embryonic neural stem cells *in vivo*. We show that the actual distribution of measured gene expression ratios for pairs of neural stem cells is much broader than that predicted from our sampling effect model.

**Conclusion:**

Our results confirm that significant differences in gene expression levels exist between phenotypically identical cells *in vivo*, and that these differences exceed any noise contribution from global mRNA amplification.

## Background

As our ability to investigate molecular mechanisms in biology at finer resolutions improves, there is increasing interest in generating reliable gene expression profiles for smaller biological samples, down to the level of the single cell and potentially subcellular compartments. Single-cell gene expression profiling provides a powerful tool to analyze the composition of complex cell populations [1]. There are many contexts in which the focus is shifting towards understanding the cellular networks of individual cells [2,3] and the similarities and differences between individual cells at the transcriptional and translational level [4,5].

Limitations to the sensitivity and resolution of current technologies for studying gene expression mean that when using samples as small as those generated from single cells we are inevitably faced with amplifying cellular mRNA. Although the most common method for evaluating large-scale gene expression is through microarray technology [6,7], the problem will be the same for any experimental method that requires transcript amplification to produce useful quantities of material to be analyzed, including real-time PCR and serial analysis of gene expression (SAGE) [8]. The amplification stage may, however, introduce significant distortions in the measured gene expression levels, especially for genes with small numbers of transcripts in the material under study. This distortion is introduced by sampling effects that arise from inefficiencies in the processes of copying and amplifying the original mRNA pool.

In a complex mRNA population with small absolute numbers of individual transcripts, such as that from a single eukaryotic cell, sampling effects can result in only a subset of the population of starting RNA molecules being represented in the final amplified population. This is particularly problematic for low copy number transcripts in single cell samples: in the first step of the process, reverse transcription may fail for a small proportion of the original mRNA molecules, which would therefore be eliminated from subsequent amplification and detection. For genes with only a small number of transcripts in the starting material, this will create a variable (assuming the failures are random) distortion in the relative representation of transcript abundances in the final experimental sample, potentially leading to the absence of such low abundance transcripts in the final amplified population. The first round of PCR amplification will have a similar effect, and subsequent rounds will have effects of diminishing importance, in terms of complete dropout of low-abundance transcripts.

The overall effect of random dropouts of low abundance transcripts from amplified single cell cDNA populations would be that random sets of transcripts would be called as absent in different cells. Observations consistent with such sampling effects in single cell expression analysis have been reported previously, leading to the proposal that there are limits to the reliable detection of gene expression from small samples[9]. For example, one estimate is that there is a lower limit of 80 copies of a single mRNA per cell for detection of two-fold differences between samples[10]. Despite these empirical predictions, the nature and significance of sampling effects for single cell expression profiling have not been systematically studied to date.

The magnitude of the overall sampling effect will, in theory, depend on two factors: the transcript abundance distribution, which is the variation of transcript number among genes being expressed in a cell (and in particular the relative numbers of genes with low transcript numbers); and the copying and amplification efficiencies for conversion of the original population of mRNA molecules into DNA or RNA detectable by the expression profiling platform in use. We have previously demonstrated that a global polyadenylation and PCR-based amplification technique generates reliable data from picogram amounts of RNA [11], although that study did not measure the efficiency of conversion of original mRNA transcripts into cDNA copies. The copying and amplification efficiencies can be estimated from experimental data. However, the estimation of the transcript abundance distribution poses two distinct problems: knowing the form of the distribution; and evaluating the shape and scale parameters for the distribution.

There are conflicting reports of the transcript abundance distribution in a typical eukaryotic cell, ranging from a distribution with a median value for mRNA transcript copies per gene of less then one [12], to a distribution with a median of approximately 100 copies [9]. The difficulty is that, in general, the transcript abundance distributions of real single cells are not known but are inferred from population measurements (for estimates from cDNA library and SAGE library sequencing of whole tissues, see references [8,13,14]). Based on published data [9,12], a simple approximation is that the transcript abundance distribution is log-log-normal, as this distribution captures certain key features of our current understanding of the single cell transcript abundance distribution: there is a high number of genes with transcript abundances lower then 10–20 and relatively few genes with high transcript abundances (exceeding 1000 copies per cell). For the purposes of modeling single cell expression data we use that distribution for this work, with the additional assumption that such a population-based distribution is reflected in the underlying single cell transcript abundance distributions.

The purpose of this work was to systematically evaluate the presence and significance of sampling effects in PCR-based global amplification-based single cell expression profiling. We investigated whether observed variations in gene expression levels in single cell samples could be artifacts of the experimental method, how much sampling effects contribute to variability in single cell expression measurements, and, finally, if global amplification techniques can be reliably used for the detection of differences in gene expression among single cells. We conclude that significant differences in gene expression levels exist between phenotypically identical cells *in vivo*, and that these differences exceed any noise contribution from global mRNA amplification.

## Results

### Conceptual approach

Variation in microarray-based, single cell expression measurement is contributed to by (i) technical noise intrinsic to the microarray platform; (ii) sampling effects caused by non-representative amplification of low abundance mRNA transcripts; and (iii) real differences in gene expression levels between two samples. It was not possible to measure directly sampling effects in pairwise hybridizations comparing gene expression between individual cells (cell-vs-cell hybridizations), because at the outset of this research we did not know if two phenotypically identical cells were identical at the transcriptional level. However, it is possible to estimate and model sampling effects using computational methods.

**Figure 1:**
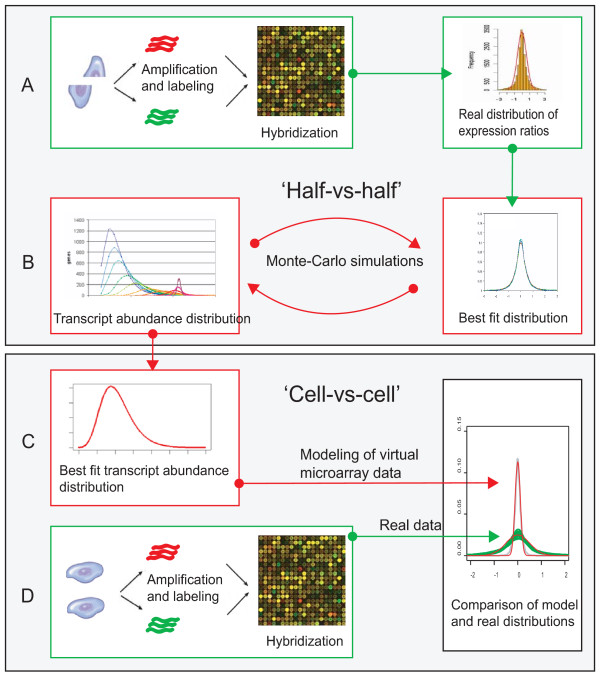
**Experimental design and computational approach to calculate the contributions of sampling effects to single cell microarray data**. **A**. Generation of experimental microarray data from single cell RNA split into two equal parts (half-vs-half microarray hybridizations); **B**. Monte-Carlo simulation of half-vs-half microarray data, based on transcript distributions and estimation of the most likely transcript distributions best fitted to observed microarray data; **C, D**. Monte-Carlo simulation of cell-vs-cell microarray data based on the most likely model transcript distribution estimated as described above. Generation of experimental microarray data from pairwise comparisons of single neural stem cells RNA samples. Finally, simulated gene expression ratio distributions were compared with experimental microarray data comparing gene expression between pairs of neural stem cells.

To do so, we performed the following steps to identify the sources of variation and noise in single cell microarray-based expression profiling (Fig. 1):

1. Estimation of technical noise;

2. Measurement of the efficiency of the amplification technique;

3. Generation of experimental microarray data comparing gene expression between two half-samples from the same cell (half-vs-half hybridizations);

4. Estimation of the single cell transcript abundance distribution from Monte Carlo simulations, by finding the distribution with the best fit to experimental half-vs-half data (measured in 3);

5. Simulation of gene expression data including observed sampling effects for pair-wise cell-vs-cell hybridizations using findings from 1–4;

6. Generation of actual microarray data for cell-vs-cell hybridizations and comparison with simulated cell-vs-cell data.

### Estimation of technical noise

**Figure 2:**
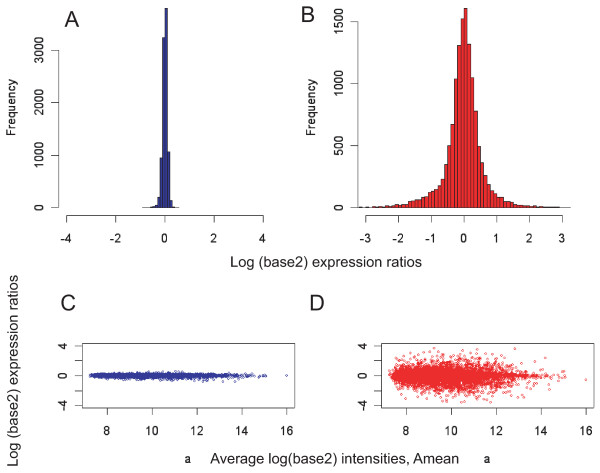
**The distribution of observed expression ratios from two, independently-amplified replicates is significantly narrower than that observed from a split cell comparison**. **A, B **– Frequency histograms for averaged log(base2) expression ratios calculated from two dye-swapped replicate hybridizations from whole cortex total RNA (A) and from two dye-swapped split cell (half-vs-half) hybridizations (B). See below for further details of the split cell hybridization procedure. **C, D **– Plots of intensity versus expression ratios (MA-plots) for averaged microarray data obtained in the two sets of dye-swapped replicate hybridizations shown in A and B. Note that the spread of expression ratios is markedly wider in the split cell comparisons than in the replicate hybridizations.

To calculate technical noise we conducted replicate hybridizations of mRNA isolated from the developing mouse dorsal forebrain (mouse neocortex at embryonic day 11.5). As our purpose was to calculate technical noise, we wished to avoid sampling effects in the initial reverse transcription and PCR steps. Therefore, we used a sufficiently high amount (10 ng) of total RNA in the initial reverse transcription and first 10 cycles of PCR. Reverse-transcribed cDNA was then used for an initial 10 cycles of PCR amplification, following which 1/200^th ^of that PCR product was used for a further 28 cycles of PCR amplification. Two replicates were then labeled and co-hybridized on expression arrays. The data shown represent the average data from two dye-swap hybridizations plotted as a frequency histogram of log(base2) expression ratios and a typical microarray plot where log(base2) expression ratios are plotted against average log(base2) signal intensities (Fig. 2). The distribution of expression ratios demonstrated very low variability between two independently amplified replicates, with standard deviation (SD) values varying from 0.10 to 0.13 (n = 4). The measured expression ratio distribution for technical noise (SD = 0.11) was used in subsequent model computations. For comparison, a typical frequency histogram and microarray plot for a comparison of gene expression between two halves of the same cell (half-vs-half, or split-cell hybridization) are also shown (Fig. 2; see below for further details).

### Estimation of global amplification efficiency

To estimate the overall efficiency of transformation of the original mRNA sample into detectable PCR product, it is crucial to know both the efficiency of reverse transcription of mRNA into single-stranded cDNA and the efficiency per round of the subsequent PCR amplification process. Distortion of the original mRNA profile is most severe for losses during the early stages of this process, and we estimate that after seven cycles of PCR amplification that the further impact on the expression profile is negligible. From measurements of cDNA mass during the process (see Additional data files 1, 2, 3); we estimated that the initial copy step (mRNA to polyadenylated single-strand cDNA) was 94–96% efficient, and that each subsequent PCR cycle was >99% efficient. Combining these values, and imposing a seven cycle limit on the effect on overall efficiency, gives us a value of 90% for the overall efficiency of transformation of the original mRNA sample into detectable PCR product.

### Generation of data comparing gene expression between two halves of the same cell: 'half-vs-half' comparisons

The first step in the process to estimate the typical single cell transcript abundance distribution was the generation of expression data comparing two halves of a single cell. The key to this approach was that by comparing data from two halves of the same cell we guarantee that the two samples were drawn from the same transcript abundance distribution (by definition), and that this is true for both the experimental and the simulated data (see below). Thus, the model abundance distribution should then apply when the same cell types are used in a straightforward two-cell comparison.

To generate these data, total RNA from four individual neocortical progenitor cells was split into two halves and each half independently amplified. Each set of two paired half-cell cDNAs was compared to one another in two dye-swapped replicate comparisons in a series of eight microarray hybridizations. These data were then used to calculate the average gene expression ratio distribution when comparing two halves of the same cell (see Fig. 2 for example).

### Modelling transcript abundance distribution

In this study, we assume that the general form of the transcript abundance distribution for single cells is log-log-normal described by the equation:

$$g=\frac{1}{x\sigma \sqrt{2\pi }}\times \exp(-{\left(\ln x-\mu \right)}^{2}/{\left(2\sigma \right)}^{2}),$$

where ***g ***is the number of genes with ***t ***transcripts, ***t ***is the transcript number and ***x *= *ln(t)***. The scale parameter ***μ ***determines the most common transcript number, and the shape parameter σ determines the width of the peak about the most common transcript number.

In order to estimate the most likely transcript abundance distribution for the single cells analysed here, we varied the scale and shape parameters of the distribution to better fit the experimental distribution observed in the 'half-vs-half' microarray data. There are some additional constraints: the number of active genes in the cell (i.e. those with transcripts present) and the total number of transcripts in the cell. Clearly these must lie within biologically meaningful limits. The consequence of this was that we could define a model cell as having ***G ***active genes and ***T ***total transcripts, and then for a given value of the scale parameter ***μ ***there was only one value of the shape parameter σ that would populate the model cell with the correct numbers of genes and transcripts, giving a specific transcript distribution. This simplifies matters somewhat as we now do not have to search all possible values of ***μ ***and σ in order to cover the feasible range of realistic model cells. For each model cell we used values of ***μ ***such that the most common transcript number varied over a range from ~2–3 to ~100. Values outside this range are likely to represent unfeasible distributions.

Preliminary analysis of the experimental data from our pairs of real single cells showed an average of ~13,000 genes per experiment with a measurable gene intensity ratio, with ~23,000 probes on the microarray. We created a range of 15 model cells using gene numbers between 10,000 and 20,000 and total transcripts between 500,000 and 2 million (see Additional file 4). In the event, this empirically chosen range was sufficient. For each model cell we created ~10–20 specific distributions for values of ***μ ***in the range 0.50 – ~2.00 (there is an effective upper limit for ***μ ***for each model, depending on the transcript number, where the peak becomes too sharp to model effectively), giving us a set of 205 specific distributions. Each specific distribution consisted of a vector of pairs of values: a gene transcript number and the number of genes with that number of transcripts (see Additional data file 4).

### Estimation of parameters of transcript abundance distribution fitted to half-vs-half microarray expression data

We ran a simulation for each model transcript distribution to create simulated half-cell log intensity ratio distributions. The simulated half-cell log intensity ratio distributions for each transcript distribution for the various model cells were then compared with the real log intensity ratio distribution from the experimental comparison of two half samples of mRNA from the same cell. To measure the fit between the real and simulated data we used the root mean square (rms) difference on the vertical axis of the intensity distribution over the log ratio range -3.0 to +3.0 averaged over the runs for each distribution.

**Figure 3:**
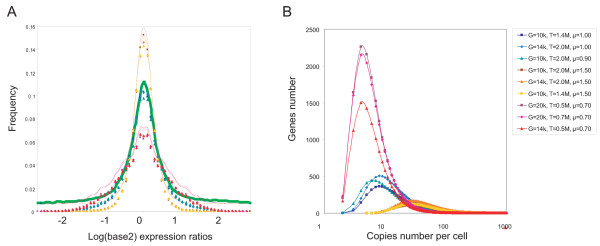
**Split cell expression comparisons: fitting modeling predictions to observed data**. **A**. Simulations of microarray expression data comparing two halves of the same cell were performed for a range of transcript abundance distributions and compared to observed data. The experimental log intensity ratio distribution (broad green curve) for two halves of a neural stem cell is shown, and superimposed on this are data for (i) the three transcript distributions that are closest to the real data (blue-green points), and the three worst fit distributions for (ii) high **μ **values (yellow-brown), and (iii) low **μ **values (red-pink). X-axis: log intensity ratio (data divided into 0.10 log unit bins); y-axis: proportion of genes in each bin. **B**. Model transcript distributions for the best and worst fit cases, colour coded as in A. The distributions that fit the observed data best (blue-green) segregate from the poorer fit distributions. X-axis: transcript copy number for a gene; y-axis: the number of genes with a given transcript copy number. The legend shows the independent parameters determining each model distribution: **G **= active genes, **T **= total transcripts, **μ **= scale value of log-log-normal distribution.

**Figure 4:**
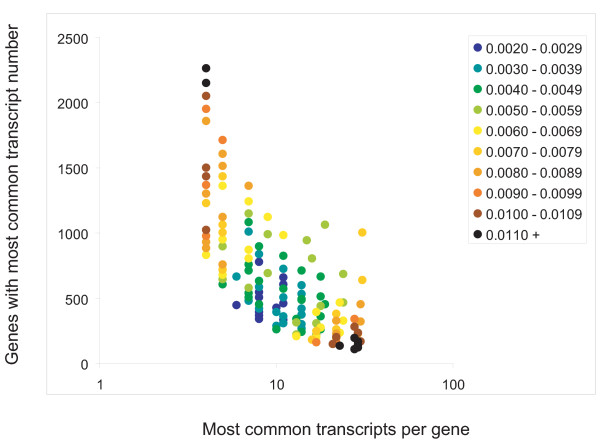
**The positions of transcript distribution peaks for all split-cell simulations, colour-coded according to the fit with the real data, predict an average number of transcripts per gene**. This figure summarizes the data from all the split-cell simulations by representing each model transcript abundance distribution as a single point at the position of its peak. The points are color coded according to the *rms *(root mean square) distance from the real data (blue, best fit; brown-black, worst fit), and clearly show the clustering of the best-fit distributions in one region of the graph. X-axis: transcript copy number for a gene; y-axis: the number of genes with a given transcript copy number.

The closer the fit between the real and simulated microarray data, the closer the model distribution will be to the transcript distribution in the real cell (Fig. 3A). The basic cell model (gene and transcript number) varied quite widely over the best fit distributions but the best fit distributions themselves showed considerable consistency, with ***μ ***values around 1.00. Plotting the peaks of all these distributions shows even more clearly the strong correspondence between the distributions and fit with the two half-cell real data (Fig. 4). From this we conclude that although we cannot determine the basic cell model with any accuracy, we can estimate the probable transcript distribution for cells of the type in this study as being that for which the most common transcript number ~8 and the number of genes with that transcript number ~500.

### Single neural stem cells demonstrate considerably greater differences in gene expression than predicted by a sampling model

If single phenotypically identical cells are very similar at the transcriptional level, we would expect that the simulated microarray data would approximate results produced from real expression data. To test this, we generated data comparing expression between twelve single, murine embryonic neural stem cells in a series of pairwise hybridizations that were repeated as dye-swapped replicates. These cells were chosen because they are phenotypically identical, neurogenic neural stem cells isolated from the same region of the mouse neocortex at a single developmental timepoint at which they have a cell cycle length of approximately 12 hours.

**Figure 5:**
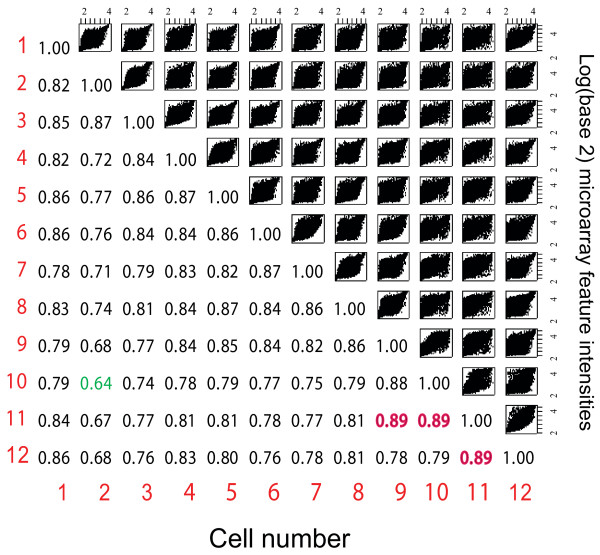
**Pairwise comparisons of gene expression among a set of 12 single neural stem cells demonstrate considerable differences in gene expression**. A matrix of scatterplots of averaged intensity values from pairs of dye-swapped hybridisations comparing individual pairs of neural stem cells is shown. Numbers in the table indicate the Spearman correlation coefficients between expression levels for pairs of cells; the lowest correlation coefficients are green and highest are red.

**Figure 6:**
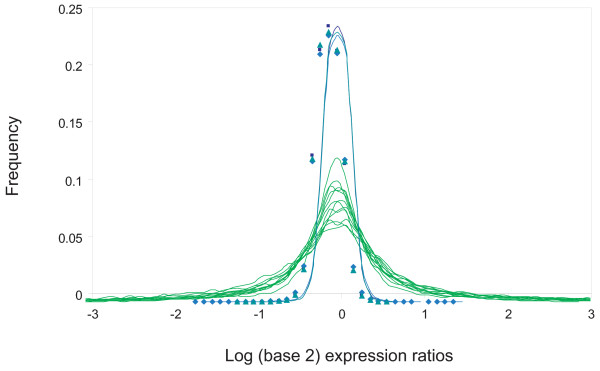
**Observed data comparing two phenotypically identical cells diverge markedly from modeling predictions of such a comparison**. Simulations of expression data comparing two identical cells were performed for the most likely transcript abundance distributions indicated by the half-vs-half (split-cell) simulations (curves with data points shown), and compared to actual data from pairwise comparisons of single neural stem cells (thin green curves). The distributions for the real cells are all considerably wider than for the model cells, showing real variation of gene expression that cannot be explained by a combination of technical noise and the sampling effect. X-axis: log intensity ratio (data divided into 0.10 log unit bins); y-axis: proportion of genes in each bin.

Analysis of the gene expression data from those cells identified significant transcriptional differences among these cells (Fig. 5). Comparing these data from real cells with our simulated microarray data found that the datasets were notably different in the spread of expression ratios (Fig. 6). This can only reasonably be explained on the basis that there are widespread and significant variations in individual gene expression levels amongst real cells of this type even though they are phenotypically identical.

## Discussion

The main findings of this study are that the contribution of sampling effects to observed single cell expression data is likely to be minor and that substantial transcriptional differences exist between phenotypically identical cells. This indicates that one can generate reliable gene expression profiles from single cells using microarrays to interrogate globally amplified RNA populations. However, the considerable variation in gene expression levels between similar cells is likely to dictate that relatively high numbers of cells would need to be analysed to robustly identify significant and consistent differences in gene expression between cell populations. Alternatively, these findings argue that single cell expression profiling will be particularly useful for identifying absolute differences in gene expression between cell types.

A second implication of this study is that one important limit on the use of amplification techniques for single cell expression profiling is that if amplification efficiency drops significantly below 90% then the sampling effect may considerably distort the measured expression profile. If the goal of a given investigation is to use sub-picogram samples of RNA or to measure very rare transcripts, than the efficiency of amplification becomes even more important. There are several amplification techniques that have been used for single cell expression profiling [11,15-21]. One promising technique for mRNA amplification from individual cells, which combines global exponential and linear amplification, has been shown to produce very low levels of noise and highly reproducible data and may limit the significance of sampling effects when profiling rare transcripts [22].

### The majority of transcripts are present at relatively low abundance

Our results demonstrate that the actual transcript abundance distribution for the tested cell type has a peak at approximately 5–20 copies per gene. We recognize that our experiments are based on a particular type of mouse neural stem cell, but in the absence of any reason to suppose that the transcript distributions of most other cell types are radically different from this, we believe the result should generally apply to expression experiments performed on a wide range of cell types.

Previous work has demonstrated that the distribution of mouse transcript abundances in E12.5 embryonic, placental, and cultured embryonic and trophoblast stem cells are highly similar, suggesting that such distributions are not heavily skewed according to tissue structure or function [12]. That study estimated that the percentage of transcripts present at less than an average of one copy per cell ranged from 40.1 to 48.2% in the four tissues [12]. We propose that the typical transcript abundance distribution in eukaryotic cells is log-log-normal, consistent with previously published results in which the shape of transcript abundance distribution appears to be log-log-normal [9,12]. Although our method did not allow us to discriminate between different models of overall gene and transcript numbers in the cell, we believe it strongly suggests that more then 85% of transcripts are present in relatively low copy numbers (less then 100 copies per cell).

### Phenotypically similar cells demonstrate variability within population

We compared gene expression among twelve phenotypically identical neural stem cells randomly taken from the developing mouse neocortex. At this stage of development the population of stem progenitor cells is expected to be homogeneous [23,24], although these cells are not synchronized with respect to their position in the cell cycle. Therefore, we are confident that the observed diversity of gene expression does not reflect cellular heterogeneity. Similar and even higher diversity has been found for other cell types, including neurons (our unpublished data), and again this seems to reflect real differences in gene expression levels of individual cells.

One possibility is that the difference in cell expression levels profiles we discovered could be a result of stochastic fluctuations of mRNA levels and to be an intrinsic characteristic of the cell's behaviour. The variation in the transcript levels between homogeneous, phenotypically identical cells remains undefined, but growing evidence indicates that phenotypically similar cells are not identical at the transcriptional level. Insight into the variability of the gene expression profiles of single cells has been obtained using a number of technical approaches, incuding microarray analysis following linear T7-based amplification [16,25], multiplexed FISH (fluorescence in situ hybridization) [26] and quantitative PCR [27]. Transcriptional bursting has been observed in *Escherichia coli*, in which protein levels have very little correlation with mRNA levels, particularly for younger cells [28], as well as *Dictyostelium *[29] and mammalian cells [30]. In mammals, the study of expression levels of several genes in individual mouse pancreatic islet cells by real-time PCR revealed high heterogeneity within the population of tested cells [27]. Overall, those findings are consistent with a model for cellular phenotypes that are underwritten by transcriptional programs that appear inherently noisy when total cellular transcript levels are measured at the single cell level.

It has been suggested that because in the individual cell the transcriptional machinery is controlled by a relatively small number of transcription factors, it may result in stochastic behavior in gene activity. Our current results revealed that the majority (44%) of genes are represented by limited number of mRNA copies (less 25), and this may account for the large cell-to-cell variations in mRNA copy number that we have observed.

## Conclusion

We have addressed the degree of transcriptional variation between phenotypically identical cells by using a simple and informative approach to estimate the sampling effect introduced by single cell cDNA amplification and expression profiling. Comparing those simulated data with data generated from single neural stem cells confirmed that sampling effects do not impede our ability to extract reliable gene expression profiles from single cells and that significant differences in gene expression levels exist between phenotypically identical cells.

## Methods

### Real-time PCR

Each real time PCR mix contained 2.8 ml water, 2 ml template, 0.2 ml of each primer (10 mM) and 5 ml 2× Master mix (DyNAmo Capillary SYBR Green qPCR Kit, Finnzymes). Real-time PCR was performed in a LightCycler (Roche Diagnostics) according to DyNAmo Capillary SYBR Green qPCR protocol. Ct values were determined using the maximum second derivate function in the LightCycler software (Roche Diagnostics). Generation of PCR products was confirmed by melting curve analysis and gel electrophoresis.

### Global polyadenylated PCR amplification

Neural stem cells were obtained from dissections of the developing mouse neocoretex at day embryonic 11.5. Tissue was dissociated to a single cell suspension using papain (Worthington Biochemical Corporation) and single cells were picked by hand using glass micropipettes, washed in PBS and placed in PCR tubes with cell lysis buffer following by global polyadenylated PCR amplification as described [5]. PCR products were purified with the CyScribe GFX Purification kit (Amersham Bioscience – GE Healthcare) and labeled with Cy3/Cy5-modified dCTP using Klenow DNA polymerase (BD Bioscience). For microarray analysis of two halves of the same cell, the cell was placed in 9 ml of ice-cold stock buffer as described above, incubated for 2 min, then the lysate was divided into two parts of 4.5 μl each for global polyadenylated PCR amplification.

### Microarray hybridization

Expression microarrays containing 23232 65-mer oligonucleotides (Sigma-Genosys) were printed on Codelink slides (Amersham). Hybridized arrays were scanned with an Axon Instruments microarray scanner at a resolution of 10 μm at maximum laser power and photomultiplier tube voltage of 60–80%. Image analysis and feature analysis were performed with GenePix Pro 4.0 (Axon Instruments, Inc.)

### Statistical methods

All statistical analysis of microarray data was conducted using the R environment [31] and the R package 'Statistics for Microarray Analysis'[32]. Data normalization was performed using scaled loess normalization in the Limma package [33]. The most variable genes were detected with the Maanova package [34].

### Computer simulations

We used our model cell transcript distributions to perform Monte Carlo simulations of microarray experiments comparing samples from two halves of the same cell and from pairs of identical cells. In each case we created computer representations of each gene and transcript in the model distribution and used random numbers to simulate a 6% failure rate during the initial copying stage and a 1% failure rate over 7 rounds of PCR. Each transcript was treated independently of the gene it came from and which sample it was in. In addition, for the two half-cell simulation, we used the random numbers to simulate splitting the sample in two before copying and amplification. This gave us an effective amplified transcript concentration for each gene in each sample, from which, with the addition of a random value for each gene in the range -0.20 to +0.20 to represent technical noise, we generated a set of log intensity ratio values for each gene in the experiment. This was transformed into a log intensity ratio distribution by summing the log-ratio values for individual genes over 0.10 log unit bins and normalizing for the number of genes in the data set. Simulations were repeated 10 times for each transcript distribution in both experiments. For further details, see Additional file 4.

## Authors' contributions

TS and FJL designed the study. TS carried out the experimental research, ran preliminary analytical modeling in R-language (confirmed by simulations and not included in the paper). MJG devised and ran the Monte Carlo computer simulations and contributed parts of the text. TS and FJL wrote the manuscript. All authors read and approved the final manuscript.

## Supplementary Material

Additional file 1 Figure legends to supplementary figures. Figure legends. Click here for file

Additional file 2 Supplementary figure 1. Electropherogram of purified mRNA. Click here for file

Additional file 3 Supplementary figure 2. Ct values of Rps17 gene are proportional to total amounts of PCR-amplified DNA regardless of number of PCR cycles. Click here for file

Additional file 4 Data supplement on calculation of RT-PCR and computer modelling procedures Descrition: Data.
Click here for file
