# Supplementary material for: Modelling and measuring single cell RNA expression levels find considerable transcriptional differences among phenotypically identical cells
Source: BMC Genomics. 2008 Jun 3;9:268. doi: 10.1186/1471-2164-9-268 (PMC2429916; doi:10.1186/1471-2164-9-268)
Supplement: Additional file 1 — Figure legends to supplementary figures. Figure legends. [file 1471-2164-9-268-S1.doc]

Supplementary figure 1. Electropherogram of purified mRNA

Total neocortical RNA was isolated from 4 mouse embryos (11.5 days of neonatal development) by Trizol method. mRNA was purified from total RNA using Poly(A)Purist Kit (Ambion Inc.) Aliquot of mRNA sample was analyzed using RNA 6000 Nano Assay (Agelent Bioanalyser, Agelent Technology). There is no peaks correspondent to contamination with ribosomal RNA (18S and 28S, blue). Median of transcript’s size distribution on electropherogramm is approximately 1,000 nt (red). Narrow peak at the beginning of electropherogramm is internal marker.

Supplementary figure 2. Ct values of Rps17 gene are proportional to total amounts of PCR-amplified DNA regardless of number of PCR cycles

*Triangles*- dsDNA obtained in one cycle of GA amplification; *circles* – dsDNA resulting from 30 cycles of GA amplification. ****Real-time PCR was performed in a LightCycler (Roche Diagnostics) according to DyNamo Capillary SYBR Green qPCR protocol. Ct values were determined using the maximum second derivate function in the LightCycler software (Roche Diagnostics). Formation of PCR products was confirmed by melting curve analysis and gel electrophoresis.****
