# Supplementary material for: Modelling and measuring single cell RNA expression levels find considerable transcriptional differences among phenotypically identical cells
Source: BMC Genomics. 2008 Jun 3;9:268. doi: 10.1186/1471-2164-9-268 (PMC2429916; doi:10.1186/1471-2164-9-268)
Supplement: Additional file 4 — Data supplement on calculation of RT-PCR and computer modelling procedures Descrition: Data. [file 1471-2164-9-268-S4.doc]

**Data supplement – Subkhankulova, Gilchrist and Livesey**

**1. Calculation of the efficiency of cDNA reverse transcription and polyadenylation processes**

Total RNA was isolated from mouse embryos at age E11.5 using Trizol reagent (Invitrogen Life Technologies). mRNA was purified from total RNA (Poly(A)Purist, Ambion) followed by quality monitoring with Agilent 2100 Bioanalyzer (Agilent Technologies). An electropherogram demonstrated that mRNA had no contamination with ribosomal RNA and that the median transcript size was approximately 900 nt (Figure 1). 1772 ng of mRNA was reverse transcribed and polyadenylated according to protocol (Material and Methods). To remove salts, enzymes, free primers and nucleotides the polyadenylated cDNA (polyA-cDNA) was purified with CyScribe Purification Kit (GE Healthcare). The concentration of the purified polyA cDNA was measured with a NanoDrop Spectrophotometer (NanoDrop Technologies) and used for the calculation of input polyA-cDNA in quantitative PCR.

We assumed that the concentration of cDNA for the abundant transcript ribosomal protein S17 (rps17) was proportional to polyA-cDNA concentration before and after purification. Therefore for each dilution of cleaned polyA-cDNA (6.3, 3.15, 1.58 and 0.315 ng/µl) we calculated by real-time PCR the Ct values for rps17 mRNA/cDNA and constructed a calibration curve for rps17 Ct values against the concentration of polyA-cDNA (Plaffi, 2001). From the calibration curve we obtained:

Ct=15.592-1.408*log(C)

where C is unknown concentration of polyA-cDNA; Ct is the Ct value for rps17 gene determined using the maximum second derivate function in the LightCycler software (Roche Diagnostics). Hence the concentration of polyA-cDNA before purification was calculated as:

The expected maximal polyA-cDNA yield was calculated if all mRNA transcripts were converted into cDNA, with the assumptions that the size distribution of polyA-cDNA corresponded to that of the initial mRNA population, with the additional polyA tailing length of 75-125nt (added by recombinant terminal transferase). In additional experiments we confirmed that polyadenylation increased an average molecular weight of cDNA copies in 1.2 fold with good agreement with theoretical calculations (Table 1).

**2. Calculation of the efficiency of the first PCR cycles**

We confirmed that rps17 cDNA is amplified in proportion to the total increase of cDNA products amplified by the global polyadenylation (GA) amplification technique (Figure 2). Therefore, rps17 gene was used for calibration of GA PCR yields when it was not possible to measure an absorbance from DNA samples of low concentration. To validate the efficiency of the PCR steps of the GA amplification technique, 100pg of mRNA was amplified for 10 PCR cycles in four parallel reactions, and total yields were extrapolated from Ct values for the rps17 transcript obtained from real-time PCR (Table 2).

The maximal theoretical yield in 10 cycles of GA amplification starting with 100pg of mRNA at the exponential phase of PCR was calculated as 118 ng (Table 2). Therefore the efficiency of total procedure including cDNA synthesis, polyadenylation and 10 cycles of exponential amplification was 87%. The efficiency of exponential amplification was calculated by deduction of the efficiency of reverse transcription (96%) from overall efficiency (87%) resulting in 90.6% efficiency for 10 PCR cycles, which is the equivalent of 99.0% efficiency for each PCR cycle (Table 2).

Therefore, we lost 4% of the starting population of mRNA transcripts in the initial stage of reverse transcription/polyadenylation with subsequent loss of approximately 1% of dsDNA copies in each exponential PCR cycle. We demonstrate in the current paper that sampling effects are most crucial for rare transcripts with number of copies less then 100/cell. The maximal theoretical increase in the number of DNA molecules during 7 exponential PCR cycles is 2^7^ or 128-fold. Therefore after 7 PCR cycles even very low abundance transcripts are not subject to strong sampling, and the overall efficiency of method is no less then 90%. This figure means that during global polyadenylation amplification the loss of original mRNA transcripts does not exceed of 1 in 10 molecules.

**3. Computer simulations**

In order to simulate microarray expression data from single cell mRNA samples, we created a range of feasible model cells and sets of related model transcript abundance distributions for each cell. Random numbers were then used to run a Monte Carlo simulation of the copying and PCR stages for the global amplification of mRNA from these model cells in order to produce simulated log intensity ratio curves that could be compared with data from experiments on real cells.

*Model cells and transcript distributions*

We created an initial range of 15 model cells using gene numbers between 10,000 and 20,000 and total transcripts between 500,000 and 2 million (see Table 3.). Whether we needed to extend this range would depend on the outcome of the computer simulations, and, in the event, the range was sufficient. For each model cell we created ~10-20 specific distributions according to the log-log-normal distribution (described in the main text) for values of μ in the range 0.50 - ~2.00 (there is an effective upper limit for μ for each model, depending on the transcript number, where the peak becomes too sharp to model effectively), giving us a set of 205 specific distributions. Each specific distribution consisted of a vector of transcript number and gene count pairs, and was stored in a database for later use.

There is a specific problem in populating the high transcript number tail of the distribution where only occasional values of t get populated with a single gene. An arbitrary upper limit of 32,000 transcripts per gene was selected, and then the area under the transcript distribution curve was integrated numerically from the upper limit downwards, instantiating a gene whenever the accumulated transcript count reached the required value. In this manner the high transcript count tail of the distribution was sparsely populated with smaller numbers of discrete genes.

*Monto Carlo simulations*

1. ‘Half-vs-half’ samples

(a) For each model cell and for each specific transcript distribution for that model (intially restricted to those with μ values in the range 0.70-1.50), the following operations were carried out to simulate the microarray comparison of two half-samples from a single cell. The transcript distribution vector was retrieved from the database and used to create a numbered set of model genes, and a numbered set of model transcripts for each model gene according to the distribution. Each transcript was given a 'presence value' = 1.0. Sample division was simulated by assigning a (pseudo-)random number to each transcript and using it to allocate the transcript to either sample 1 or sample 2, with a 50% chance of going to either sample. The initial copying stage was simulated by randomly knocking out 6% (copy failure rate) of transcripts from each sample. PCR amplification (1% failure per round) was simulated by reducing the presence value of a proportion of transcripts over seven sucessive rounds: round 1, for 1 in 100 transcripts reduce presence by a factor 0.50; round 2, for 1 in 50 transcripts, reduce presence by a factor 0.67 (1 - 1/[n + 1]), etc. The fate of any transcript was independent of which gene it belonged to. The total effective transcript level for each gene in each sample was then simply calculated by summing the presence values for all surviving transcripts in each sample for each gene, and this was used as the signal intensity value for the gene. Any gene in either sample with no transcripts left was given a notional intensity value of 0.01 to represent baseline noise and prevent divide-by-zero problems. Any gene with no transcripts left in both samples was deleted. Technical noise was added to each gene intensity value as a random number in the range -0.20 to +0.20. Finally a log intensity ratio value was calculated for each surviving gene in the two samples, and a log intensity ratio distribution was created summing the log-ratio values over 0.10 log unit bins and normalising for the number of genes in the data set. Similar runs were repeated 10 times for each transcript distribution.

(b) The simulated log intensity ratio distributions for each transcript distribution for the various model cells were then compared with the real log intensity ratio distribution from the experimental comparison of two half samples of mRNA from the same cell. This was done by finding the average absolute difference between the log intensity ratio values of the simulated and the real data for each 0.10 log unit bin over the range -3.0 to +3.0 for each model transcript distribution. The best fit model distribution was the one with the smallest rms difference with the real data. The rms difference values for the four best fit and four worst fit model transcript distributions is show in Table 4.

2. Samples from a pair of identical cells (‘cell-vs-cell’)

The transcript abundance distributions that best fit the two half-cell data were then used in a simulation of a microarray comparison of two identical cells. For eight of the best fit distributions (top 5 + 8th, 10th, 11th) the following operations were carried out to simulate the microarray comparison of samples from two identical single cells. The transcript distribution vector was retrieved from the database to create a model cell as in the previous simulation. However, in this case, two identical cells were created for the distribution, and all transcripts in both cells were allocated a different random number. With the exception of the sample divison step, which was of course omitted, the simulation was run as before, producing a simulated log intensity ratio distribution for each model, for the comparison of a pair of identical cells. The simulations could then be compared graphically with equivalent experimental data derived from pairs of morphologically similar, real cells to estimate the magnitude of the sampling effect.

*Note on Random Numbers*

A set of 2 million pseudo-random numbers was created using the C rand() function and stored as a list in a database table in the order they were generated. Random numbers for all the transcripts in a given model distribution were allocated from this list starting at a different, randomly selected point for each distribution, wrapping round the end of the set where necessary.

**Table 1. Efficiency of reverse transcription and polyadenylation steps in global polyadenylated PCR-based amplification**

| Ct value for Rps17 | Estimated concentrations of polyA cDNA, ng/µl | Dilution, times | Mean concentration of polyA cDNA (SD), ng | Starting mRNA amount, ng | Yield of PolyA cDNA (SD), ng | Expected yield of polyA cDNA, ng | Efficiency  (SD), % |
| --- | --- | --- | --- | --- | --- | --- | --- |
| 13.00 | 6.302 | 1 | 6.18(0.213)  (total volume 0.318ml) | 1772 | 1965(68) | 2045 | 96.0(3.3) |
| 14.03 | 3.032 | 2 |
| 15.03 | 1.491 | 4 |
| 17.19 | 0.3214 | 20 |

**Table 2. The efficiency of PCR cycles at exponential stage of global polyadenylated PCR-based amplification**

| Calculated yield of dsDNA, ng | Measured concentration of dsDNA, mean(SD), ng/µl | Measured yield of dsDNA, ng/µl | Efficiency of total amplification | Efficiency of 10 PCR cycles | Efficiency of single PCR cycle |
| --- | --- | --- | --- | --- | --- |
| 118 | 0.935(0.245) | 102.85 | 87% | 90.6% | 99,0% |

**Table 3. The set of 15 model cells used for computer simulations.**

| Model cells | | | | |
| --- | --- | --- | --- | --- |
| 10,000 genes  500,000 transcripts  12, µ = 0.50 - 1.60 | 10,000 genes  700,000 transcripts  14, µ = 0.50 - 1.80 | 10,000 genes  1 million transcripts  15, µ = 0.50 - 1.90 | 10,000 genes  1.4 million transcripts  21, µ = 0.50 - 2.14 | 10,000 genes  2 million transcripts  15, µ = 0.50 - 1.90 |
| 14,000 genes  500,000 transcripts  11, µ = 0.50 - 1.60 | 14,000 genes  700,000 transcripts  12, µ = 0.50 - 1.50 | 14,000 genes  1 million transcripts  14, µ = 0.50 - 1.80 | 14,000 genes  1.4 million transcripts  15, µ = 0.50 - 1.90 | 14,000 genes  2 million transcripts  15, µ = 0.50 - 1.90 |
| 20,000 genes  500,000 transcripts  9, µ = 0.50 - 1.30 | 20,000 genes  700,000 transcripts  11, µ = 0.50 - 1.50 | 20,000 genes  1 million transcripts  12, µ = 0.50 - 1.60 | 20,000 genes  1.4 million transcripts  14, µ = 0.50 - 1.80 | 20,000 genes  2 million transcripts  15, µ = 0.50 - 1.90 |

The bottom row of figures shows the number of specific distributions created for each model, and the range of values of µ for which distributions were made.

**Table 4. The model distributions with the best and the worst fit for the two half cell simulations between the model and the real data.**

| Model cell | | Distribution, µ value | Rms difference to real two half-cell data |
| --- | --- | --- | --- |
| Active genes number | Total transcript number, million |
| 10,000 | 1.4 | 1.00 | 0.0021* |
| 14,000 | 2.0 | 1.00 | 0.0022 |
| 10,000 | 2.0 | 0.90 | 0.0023 |
| 14,000 | 1.4 | 1.00 | 0.0025 |
| 10,000 | 2.0 | 1.40 | 0.0122 |
| 10,000 | 1.4 | 1.50 | 0.0131 |
| 14,000 | 2.0 | 1.50 | 0.0132 |
| 10,000 | 2.0 | 1.50 | 0.0150** |

* - rms value produces the model distribution best fit to real ‘half-vs-half’ microarray data;

** - rms value produces the model distribution worst fit to real ‘half-vs-half’ microarray data;

rms: root mean square
